# Associations between self-compassion and suicidal ideation among college students: the serial mediating roles of meaning in life and psychological resilience and the moderating role of perceived stress

**DOI:** 10.3389/fpsyg.2026.1768876

**Published:** 2026-03-13

**Authors:** Yinpin Huang, Jianbin Chen, Jing Guan, Shuyi Zhao, Deng Pan

**Affiliations:** 1 School of Foreign Languages, Jingchu University of Technology, Jingmen, Hubei, China; 2 School of Literature and Cultural Communication, Tianshui Normal University, Tianshui, Gansu, China; 3 Lanzhou Vocational Technical College, Lanzhou, Gansu, China; 4 School of Foreign Languages, Hubei University of Science and Technology, Xianning, Hubei, China

**Keywords:** meaning in life, perceived stress, psychological resilience, self-compassion, suicidal ideation

## Abstract

**Background:**

Suicidal ideation is a critical concern among college students. We examined self-compassion as a key protective quality and its links with suicidal ideation. Our model places meaning in life and psychological resilience in a serial path between these variables and treats perceived stress as a contextual factor.

**Methods:**

We carried out a survey and asked students to complete the Positive and Negative Suicide Ideation Scale, the Self-Compassion Scale, the Meaning in Life Questionnaire, the short form of the Connor-Davidson Resilience Scale and the short Perceived Stress Scale. With 905 valid questionnaires, we used partial least squares structural equation modeling to explore links among these variables.

**Results:**

Self-compassion and suicidal ideation moved in opposite directions at the direct path level (*β* = −0.139, *p* = 0.004). An indirect effect also ran through a chain that first involved meaning in life and then psychological resilience (*β* = −0.008, *p* = 0.030). The product term of self-compassion and perceived stress showed a positive link with meaning in life (*β* = 0.333, *p* < 0.001), and this link was stronger when students reported higher stress.

**Conclusion:**

Overall, self-compassion shows a protective pattern for suicidal ideation through meaning in life and psychological resilience, especially in high-stress situations. These results give useful ideas for designing campus mental health programs for college students.

## 1 Introduction

According to 2021 data from the World Health Organization (WHO), suicide now holds the third place among causes of death for people aged 15–29 worldwide (World Health Organization, 2025), up from fourth place in the 2019 report (World Health Organization, 2021). This rise highlights growing concern for young people’s health and the crucial of stronger mental health care and suicide prevention services. Luceño-Moreno et al. (2025) reported higher suicide rates among college students in recent years. Suicidal ideation, includes self-harming ideas, wishing life would end, feeling tired of living and judging one’s life as worthless (Beck et al., 1979). It represents the initial phase in the progression toward suicidal behavior, often serving as a key signal to estimate the possibility of subsequent suicide attempts or death by suicide (Liu M. et al., 2025; Luceño-Moreno et al., 2025; Riera-Serra et al., 2024). Data from Chinese college students show that both lifetime and 12-month suicidal ideation are more frequent during the COVID-19 period than before (Yao et al., 2025). Preventing suicide among college students requires addressing suicidal ideation.

Current research on suicidal ideation mainly falls into three areas. One explores links between suicidal ideation and suicidal behavior (Bryan et al., 2023; Bryan and Rudd, 2023; Liang et al., 2025). Another focuses on risk factors for suicidal ideation, including depression (Jiang et al., 2024; Kim and Kihl, 2021), anxiety (Chen et al., 2025; Yang T. Q. et al., 2023), negative life events (Marrero et al., 2024; Peng et al., 2024), and stress (Dizon and Mendoza, 2023; Hussain and Hill, 2023). The third emphasizes protective factors, including social support (Mao et al., 2022), self-compassion (Suh and Jeong, 2021), meaning in life (MIL) (Lasota and Mróz, 2021), psychological resilience (Chen et al., 2023), and mindfulness (Zhou et al., 2023). However, existing studies usually examine either risk factors or protective factors in isolation, rather than how they interact within an integrated framework. Among college students, self-compassion, MIL, and psychological resilience are all important psychological protective factors. Yet, it remains unclear whether these factors change under real-life academic and interpersonal pressures, and how stress relates to these processes. This study examines associations between self-compassion and suicidal ideation in college students through an integrated model that includes MIL and psychological resilience as protective factors and perceived stress as a risk factor. The model allows us to better understand how these factors interact and how stress moderates their effects on suicidal ideation in stressful contexts.

## 2 Literature review

Suicidal ideation comes out of a layered process that spans biological, psychological, and social-environmental domains (Kirshenbaum et al., 2024). Contemporary theories often adopt an “ideation-to-action” framework to explain this process. Within this framework, Klonsky and May (2015) describe suicidal ideation in the Three-Step Theory as a state that grows out of unbearable psychological pain together with hopelessness; suicidal ideation tends to be stronger when this pain is so intense that people feel cut off from themselves and from the world. Although suicidal ideation has many determinants, self-compassion may be crucial in understanding its formation (Liu M. et al., 2025). Self-compassion is a healthy psychological tendency in which individuals react to their own failures, pain, or perceived inadequacies with warmth, understanding, and care, includes self-kindness, common humanity, and mindfulness (Neff, 2003). This construct is thought to activate the parasympathetic soothing system, thereby alleviating self-criticism and emotional distress (Gilbert, 2005), which may therefore be linked to lower suicidal ideation. Research based on evidence has consistently backed this position. Many findings show negative associations between self-compassion and suicidal ideation (Umphrey et al., 2021; Zhang et al., 2025). The positive sides of self-compassion display protective associations with suicidal ideation (You et al., 2022), and higher self-compassion appears to serve as a buffer in relation to suicide risk (Hirsch et al., 2021). Focusing on college students, Kato (2025) reports that both positive and negative self-compassion have associations with suicide risk among college students. Djajadisastra et al. (2025) find that people with more self-compassion report less suicidal ideation even when they feel highly burdensome and experience thwarted belongingness. In summary, college students with higher self-compassion tend to report lower levels of suicidal ideation.

MIL arises from the search for existential value and life purpose (Frankl, 1962). Building on this view, Reker and Wong (1988) point out that MIL offers a strong sense of purpose and coherence. Steger et al. (2006) distinguish between two aspects: presence of meaning, the conviction that one’s life is purposeful and valuable, and search for meaning, the ongoing effort to explore and deepen one’s understanding of life’s meaning. Recent work treats MIL can soften the experience of stress and failure and shows associations with a decreased chance of suicide (Cheng et al., 2024; Guo et al., 2025). Studies with Chinese college students have shown that suicidal ideation and MIL display negative associations (Cheng et al., 2024; Diao et al., 2025; Zhou and Sun, 2025); students who feel more MIL tend to report less suicidal ideation. At the same time, self-compassion shows positive associations with MIL (He et al., 2025; Wang Z. Y., et al., 2024; Wu et al., 2022). Miao et al. (2025) report that adolescents high in self-compassion also report higher MIL, and He et al. (2025) find that such students are more able to read negative experiences in a constructive way, channel distress toward emotional well-being, and establish personal significance. Chan et al. (2022) further suggest that self-compassion supports students as they face and recover from adversity, and that this support goes together with a stronger sense of MIL and less frequent suicidal ideation. Based on this line of work, we expect that college students who report higher self-compassion tend to show a clearer sense of MIL and to report lower suicidal ideation.

Psychological resilience is the process via which people effectively adapt and adjust when facing stress and adversity (Wang and Li, 2025). Individuals with high psychological resilience show strong adaptive and recovery capacities. With this capacity, students search for practical ways to handle problems, stay in a more positive state of mind, and report fewer suicidal thoughts and beliefs. Studies shows that psychological resilience is significantly negatively correlated with suicidal ideation (Chen et al., 2023; Shu et al., 2024; Yang Y. M. et al., 2023). Groups at the highest risk for suicide often report greater psychological distress and lower resilience (Ramos-Martín et al., 2023). Kumar et al. (2022) show that psychological strengths linked to resilience, including optimism and gratitude, have negative associations with suicidal ideation in sexually assaulted female college students. Evidence also points to positive associations between psychological resilience and self-compassion (Tekinarslan and Sevi Tok, 2023; Wu et al., 2024), and adolescents with high resilience generally score higher on self-compassion (Keulen et al., 2025). Therefore, we expect that college students with stronger psychological resilience show lower reported suicidal ideation.

Recent literature frequently reports a close connection between levels of MIL and psychological resilience. Studies show positive associations between MIL and psychological resilience, and students who feel more MIL often report stronger psychological resilience (Guo et al., 2025; He et al., 2025). Existential psychology suggests (Frankl, 2006) that individuals can maintain psychological resilience by finding MIL when facing adversity. When people perceive life as meaningful, they are inclined to keep a positive attitude and adopt effective coping mechanisms, which helps them better deal with challenges. Even in extreme difficulties, individuals can find the strength to endure pain by discovering the meaning of life, thus enhancing their psychological resilience (Arslan and Yildirim, 2021; Frankl, 2006; Ma et al., 2024). Lasota and Mróz (2021) argue that psychological resilience supports the maintenance of MIL. Other studies highlight the joint contribution of MIL and psychological resilience to trauma recovery and sound psychological functioning. Yildirim et al. (2025), for example, identified MIL and psychological resilience as factors that connect fear of happiness with psychological distress, noting that higher levels of both were observed alongside better mental health. Empirical evidence also shows that MIL and psychological resilience each show negative associations with suicidal ideation. Guo et al. (2025) report that psychological resilience mediates the link between MIL and suicidal ideation, with MIL helping individuals build resilience, hold hope and purpose and handle stress and failure in ways that are associated with lower suicidal ideation. In line with this pattern, He et al. (2025) show that college students with a high sense of MIL typically report stronger psychological resilience, better emotion regulation and less suicidal ideation. Self-compassion may soften self-criticism and promote positive reframing of negative experiences, which can strengthen MIL (Chan et al., 2022; He et al., 2025). In turn, MIL may support effective coping with stress and setbacks through psychological resilience, which is linked to lower suicidal ideation (He et al., 2025). By enhancing MIL, self-compassion may therefore encourage the growth of psychological resilience, and this serial pathway can give college students psychological resources that stand against suicidal ideation. On this basis, we expect that college students who score higher on self-compassion usually show higher MIL and psychological resilience and report lower suicidal ideation.

Previous work links higher perceived stress with higher suicidal ideation (Low et al., 2023; Luceño-Moreno et al., 2025; Wu and Adamsk, 2021), but may also function as a key contextual variable that moderates the psychological processes through which self-compassion exerts its protective role. Drawing on conservation of resources (COR) theory (Hobfoll, 1989), high-stress situations lead to resource depletion and prompt individuals to rely more heavily on their core psychological resources. As an internal, actively accessible positive psychological resource, self-compassion may become particularly valuable for maintaining psychological functioning (e.g., constructing meaning, sustaining resilience) under high-stress conditions. Prior research supports this view: under high-stress conditions, such as greater exposure to childhood adversity, the positive association between self-compassion and psychological resilience becomes significantly stronger (Neff and McGehee, 2009). On the other hand, stress not only depletes resources but also directly threatens individuals’ meaning systems. Meaning maintenance theory suggests that stressful events challenge people’s existing meaning frameworks and trigger strong motives to restore and compensate for meaning (Heine et al., 2006). Self-compassion can provide a safe and accepting internal psychological space in which individuals can reexamine and integrate their experiences under stress, thereby engaging more effectively in meaning construction and restoration (Neff, 2003). Empirical evidence is in line with this account; Wu et al. (2022) demonstrate that intense stress, like burnout amid the COVID-19 pandemic, alters the link between self-compassion and MIL for college students, with stressful contexts linked to stronger positive associations. Consequently, stressful contexts activate the protective link between self-compassion and MIL.

**Figure 1:**
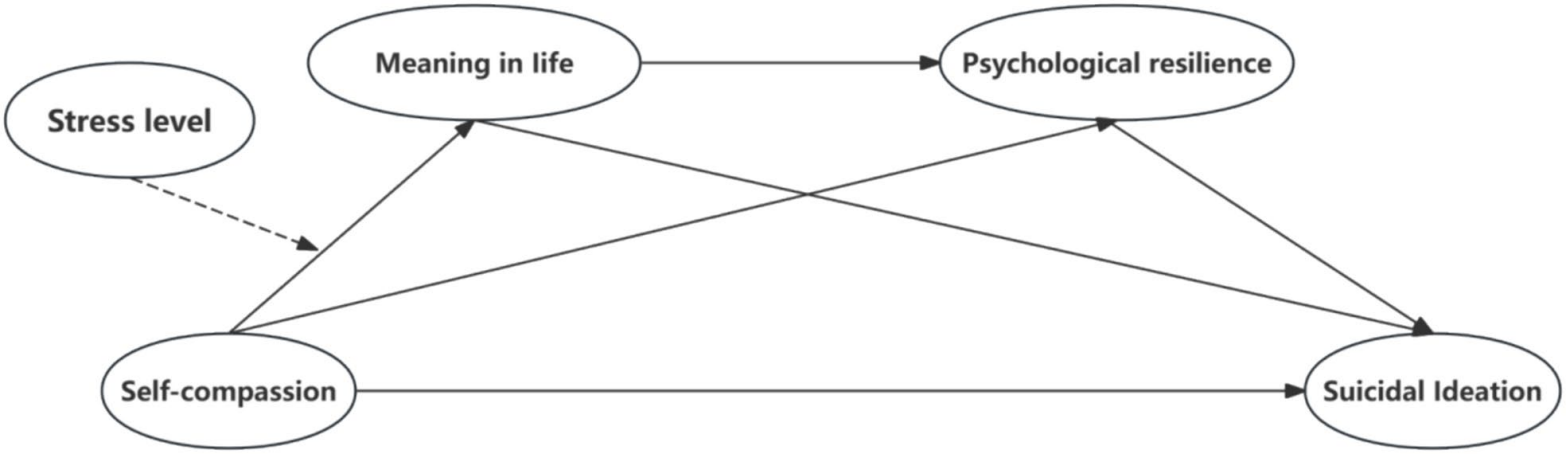
Research model.

Based on COR theory and meaning maintenance theory, we focus on associations among self-compassion, MIL, psychological resilience, perceived stress, and suicidal ideation in a sample of college students. In our conceptual model, self-compassion serves as the starting variable, MIL and psychological resilience operate as successive mediators, and perceived stress functions as a moderator of the association between self-compassion and MIL. By combining these internal psychological processes with the context of perceived stress, we build a moderated serial mediation model to describe the pattern of associations linking self-compassion, MIL, psychological resilience, perceived stress, and suicidal ideation (see Figure 1). We propose the following hypotheses:

*H1*: Self-compassion shows a negative association with suicidal ideation.

*H2*: MIL serves as a mediator in the connection from self-compassion to suicidal ideation.

*H3*: Psychological resilience mediates the association between self-compassion and suicidal ideation.

*H4*: MIL and psychological resilience act together as serial mediators in the association between self-compassion and suicidal ideation.

*H5*: Perceived stress moderates the association between self-compassion and MIL.

## 3 Methods

### 3.1 Sample source and data collection

From November to December 2025, we invited college students to take part in a questionnaire study. To select participants, we used a convenience sampling approach to distribute the questionnaires to students. Before data collection, we talked with course instructors, who presented the study in class and helped organize the survey. We then sent the questionnaires online through class group chats. We told students that taking part was voluntary, that their responses would stay anonymous and private, and that they could stop at any time; only those who agreed and gave informed consent completed the questionnaire.

We collected 932 questionnaires in total. Following previous research (Wang Z. et al., 2024), we first excluded invalid responses, such as questionnaires completed in less than 1 min, and obtained 905 valid questionnaires, resulting in a valid response rate of 97.1%. A total of 259 participants were men (28.6%) and 646 were women (71.4%), most were 18–22 years old, first- and second-year students made up 63.8% of the sample (578 students), third- and fourth-year students made up 17.0% (154 students) and 19.1% (173 students), and 69.8% of the participants came from rural areas (Table 1).

**Table 1:** Demographic characteristics.

Demographic characteristics	Category	Quantity	Proportion
Gender	Male	259	28.6%
	Female	646	71.4%
Age	18–20	299	33.0%
	20–22	288	31.8%
	> 22	318	35.1%
Grade	Freshman	289	31.9%
	Sophomore	289	31.9%
	Junior	154	17.0%
	Senior	173	19.1%
Household registration type	Urban	273	30.2%
	Rural	632	69.8%

### 3.2 Measurement tools

#### 3.2.1 Suicidal ideation

To assess suicidal ideation over the last 2 weeks, we administered the Positive and Negative Suicide Ideation Scale designed by Osman et al. (2002) and later work with Chinese college samples has provided evidence for its solid reliability (Diao et al., 2025). The scale has 14 items and includes two dimensions: positive and negative suicidal ideation. Example items are “I feel helpless about my problems and want to commit suicide.” Participants used a 5-point Likert scale to rate how often they had each thought from 1 “never” to 5 “always.” We reverse-scored the positive suicidal ideation items, so that higher total scores indicated stronger suicidal ideation. Internal consistency for the full scale was outstanding (*α* = 0.926), with subscale *α* values of 0.832 and 0.881. Confirmatory factor analysis (CFA) yielded an acceptable fit indices (*χ*^2^/df = 2.108, TLI = 0.983, CFI = 0.985, SRMR = 0.010, RMSEA = 0.035).

#### 3.2.2 Self-compassion

We used the Self-Compassion Scale developed by Neff (2003). Studies on Chinese college students have reported good reliability for this measure and have used it widely in this group (Liu M. et al., 2025). The scale contained 26 items covering six components. Example items asked how often participants responded kindly to themselves when they felt inadequate or how they approached their feelings when they were in a low mood. Example items include “I can tolerate my shortcomings and deficiencies” and “When going through difficulties, I was a bit harsh on myself.” Participants rated each item on a 5-point Likert scale. We reversed the scores for the self-judgment, isolation, and over-identification subscales so that higher total scores stood for higher self-compassion. Cronbach’s *α* for the full scale reached 0.956, and the six subscales showed *α* values of 0.793, 0.785, 0.789, 0.806, 0.787, and 0.727. CFA produced the following fit indices (*χ*^2^/df = 1.741, TLI = 0.983, CFI = 0.985, SRMR = 0.010, RMSEA = 0.029).

#### 3.2.3 Meaning in life

We used the Meaning in Life Questionnaire (Steger et al., 2006). Studies with Chinese college students report solid reliability and validity for this scale (He et al., 2025). This measurement tool contains 10 questions (e.g., “I am very clear about what makes my life meaningful”) and covers two aspects: presence of meaning and search for meaning. We used a 7-point Likert scale. Higher total scores reflected a stronger sense of MIL. The overall scale demonstrated a Cronbach’s *α* of 0.902, and the *α* coefficients for the two dimensions were 0.817 and 0.851, respectively. CFA produced the following fit indices (*χ*^2^/df = 2.565, TLI = 0.982, CFI = 0.986, SRMR = 0.039, RMSEA = 0.042).

#### 3.2.4 Psychological resilience

We used Chinese short form of the Connor-Davidson Resilience Scale modified by Wang et al. (2010). Studies with Chinese college students have reported satisfactory reliability and validity for this version (Liu J. et al., 2025; Wang and Li, 2025). The scale has 10 items, such as “I can adapt when changes occur.” Participants responded on a 5-point Likert scale from 0 “never” to 4 “always.” Higher total scores were linked with greater psychological resilience. Cronbach’s *α* for the scale was 0.887. CFA showed acceptable model fit (*χ*^2^/df = 4.344, TLI = 0.963, CFI = 0.972, SRMR = 0.015, RMSEA = 0.061).

#### 3.2.5 Perceived stress

We assessed perceived stress during the past month with the Chinese short form of the Perceived Stress Scale translated and updated by Chen et al. (2021). Research using the short form with college samples has reported satisfactory reliability and validity (DiFonte et al., 2022). The scale has 10 items, including “I felt that I was on top of things.” Participants responded on a 5-point Likert scale ranging from 0 “almost never” to 4 “always.” Elevated total scores suggested greater perceived stress. Cronbach’s *α* for the present sample was 0.906. CFA showed acceptable model fit (*χ*^2^/df = 3.798, TLI = 0.970, CFI = 0.976, SRMR = 0.015, RMSEA = 0.056).

### 3.3 Data analysis

We used SmartPLS 4.0 to conduct PLS-SEM, which has been recommended for studies that examines complex associations among psychological variables in college students (Becker et al., 2022). PLS-SEM suits samples of this size, can work with non-normal data and complex models, and needs fewer cases than covariance-based SEM (CB-SEM) for a stable ratio between participants and indicators (Hair et al., 2021; Leguina, 2015; Premkumar and Bhattacherjee, 2008). Our dataset contained 905 valid responses and a model with five constructs and 66 indicators, which fits these conditions well. With this approach, we estimated both the measurement and structural models and examined the links among self-compassion, MIL, psychological resilience, perceived stress, and suicidal ideation. The measurement model was assessed using composite reliability (CR), average variance extracted (AVE), and factor loadings, while the structural model was evaluated based on path coefficients, significance levels, and *R*^2^ values. We assessed multicollinearity using variance inflation factors (VIF) and tested indirect effects using bias-corrected bootstrapping with 5,000 resamples, with a significance threshold of 5% (*p* < 0.05).

## 4 Results

### 4.1 Descriptive statistics

Using SPSS, we obtained descriptive indices (M, SD) and Pearson r values for each main variable (Table 2). Self-compassion showed positive links with MIL and psychological resilience, and negative links with suicidal ideation and perceived stress. All correlations were significant (*p* < 0.001).

**Table 2:** Descriptive statistics.

Constructs	M ± SD	SC	SI	MIL	PR	PS
SC	2.962 ± 0.493	1				
SI	3.125 ± 0.480	−0.219^***^	1			
MIL	4.166 ± 0.953	0.555^***^	−0.170^***^	1		
PR	2.946 ± 0.509	0.279^***^	−0.215^***^	0.219^***^	1	
PS	2.938 ± 0.548	−0.305^***^	0.390^***^	−0.284^***^	−0.175^***^	1

*N* = 905, ***p < 0.001.

### 4.2 Confirmatory factor analysis

We ran CFA on the measurement model to examine construct validity and overall fit with the theoretical structure. The fit indices in Table 3 show acceptable model fit (Hu and Bentler, 1999), and we used this model for the subsequent path analyses.

**Table 3:** Model fitting index.

Fitting Index	Reference value	Fitted value
CMIN/DF	<5	1.466
GFI	>0.9	0.906
CFI	>0.9	0.967
IFI	>0.9	0.967
SRMR	<0.08	0.019
RMSEA	<0.08	0.023

### 4.3 Common method bias (CMB)

We verified CMB with Harman’s single-factor test. The first factor explained 25.14% (<40%) of the variance (Podsakoff et al., 2003), so CMB was not an issues in our investigation.

Additionally, multicollinearity was assessed at the construct level using VIF (Leguina, 2015). Values below 3.3 are usually taken as acceptable. As shown in Table 4, all VIFs lay between 1.092 and 1.174, so multicollinearity was not a significant problem. This suggests that there is no high correlation among the constructs in the model, and the results are unlikely to be affected by multicollinearity.

**Table 4:** Collinearity test.

Constructs	SC	SI	MIL	PR	PS
SC		1.174	1.111		
SI					
MIL				1.174	
PR		1.092			
PS		1.119	1.106	1.105	

### 4.4 Measurement model

Following Leguina (2015), we checked the reliability and validity of the measurement model. After careful consideration, we removed items SC1, SC20, SC23, and PR5 (Table 4). The reason for their removal is that these items had low factor loadings and negatively impacted the overall construct validity. These deletions did not alter the original dimensions or theoretical coverage of the scale, and the remaining items still adequately represent the original constructs, the remaining items and the CR values all reached acceptable levels. AVE values for each construct were above 0.50 (Table 5), which supported convergent validity. All HTMT values were below 0.85 (Table 6), and for each construct the square root of its AVE in Table 7 was larger than its correlations with the other constructs, so discriminant validity was also satisfactory.

**Table 5:** Reliability and validity.

Constructs	Items	Outer loadings	Cronbach’s *α*	CR	AVE
SC	SC2	0.698	0.956	0.960	0.509
	SC3	0.685			
	SC4	0.687			
	SC5	0.707			
	SC6	0.755			
	SC7	0.734			
	SC8	0.737			
	SC9	0.712			
	SC10	0.682			
	SC11	0.733			
	SC12	0.695			
	SC13	0.723			
	SC14	0.681			
	SC15	0.736			
	SC16	0.717			
	SC17	0.722			
	SC18	0.704			
	SC19	0.764			
	SC21	0.711			
	SC22	0.729			
	SC24	0.699			
	SC25	0.701			
	SC26	0.686			
SI	SI1	0.745	0.926	0.936	0.511
	SI2	0.697			
	SI3	0.705			
	SI4	0.718			
	SI5	0.727			
	SI6	0.706			
	SI7	0.741			
	SI8	0.715			
	SI9	0.693			
	SI10	0.759			
	SI11	0.692			
	SI12	0.713			
	SI13	0.691			
	SI14	0.702			
MIL	ML1	0.742	0.902	0.919	0.532
	ML2	0.742			
	ML3	0.739			
	ML4	0.732			
	ML5	0.719			
	ML6	0.734			
	ML7	0.734			
	ML8	0.722			
	ML9	0.691			
	ML10	0.736			
PR	PR1	0.718	0.887	0.909	0.526
	PR2	0.722			
	PR3	0.702			
	PR4	0.777			
	PR6	0.716			
	PR7	0.695			
	PR8	0.755			
	PR9	0.729			
	PR10	0.709			
PS	PS1	0.818	0.906	0.922	0.543
	PS2	0.726			
	PS3	0.723			
	PS4	0.754			
	PS5	0.742			
	PS6	0.703			
	PS7	0.697			
	PS8	0.705			
	PS9	0.702			
	PS10	0.786			

**Table 6:** Discriminant validity (HTMT criterion).

Constructs	SC	SI	MIL	PR	PS
SC					
SI	0.228				
MIL	0.594	0.186			
PR	0.594	0.238	0.592		
PS	0.329	0.425	0.312	0.194	

**Table 7:** Discriminant validity (Fornell–Larcker criterion).

Constructs	SC	SI	MIL	PR	PS
SC	***0.713***				
SI	−0.217	***0.715***			
MIL	0.554	−0.171	***0.729***		
PR	0.272	−0.221	0.219	***0.725***	
PS	−0.304	0.390	−0.287	−0.180	***0.736***

Under the Fornell–Larcker criterion, each construct’s correlations with other constructs should be lower than the square root of its AVE; the bold italic values on the diagonal represent the square root of the AVE.

### 4.5 Structural model

In evaluating the structural model, we inspected collinearity diagnostics together with the path coefficients and coefficients of determination.

#### 4.5.1 Path hypotheses

**Figure 2:**
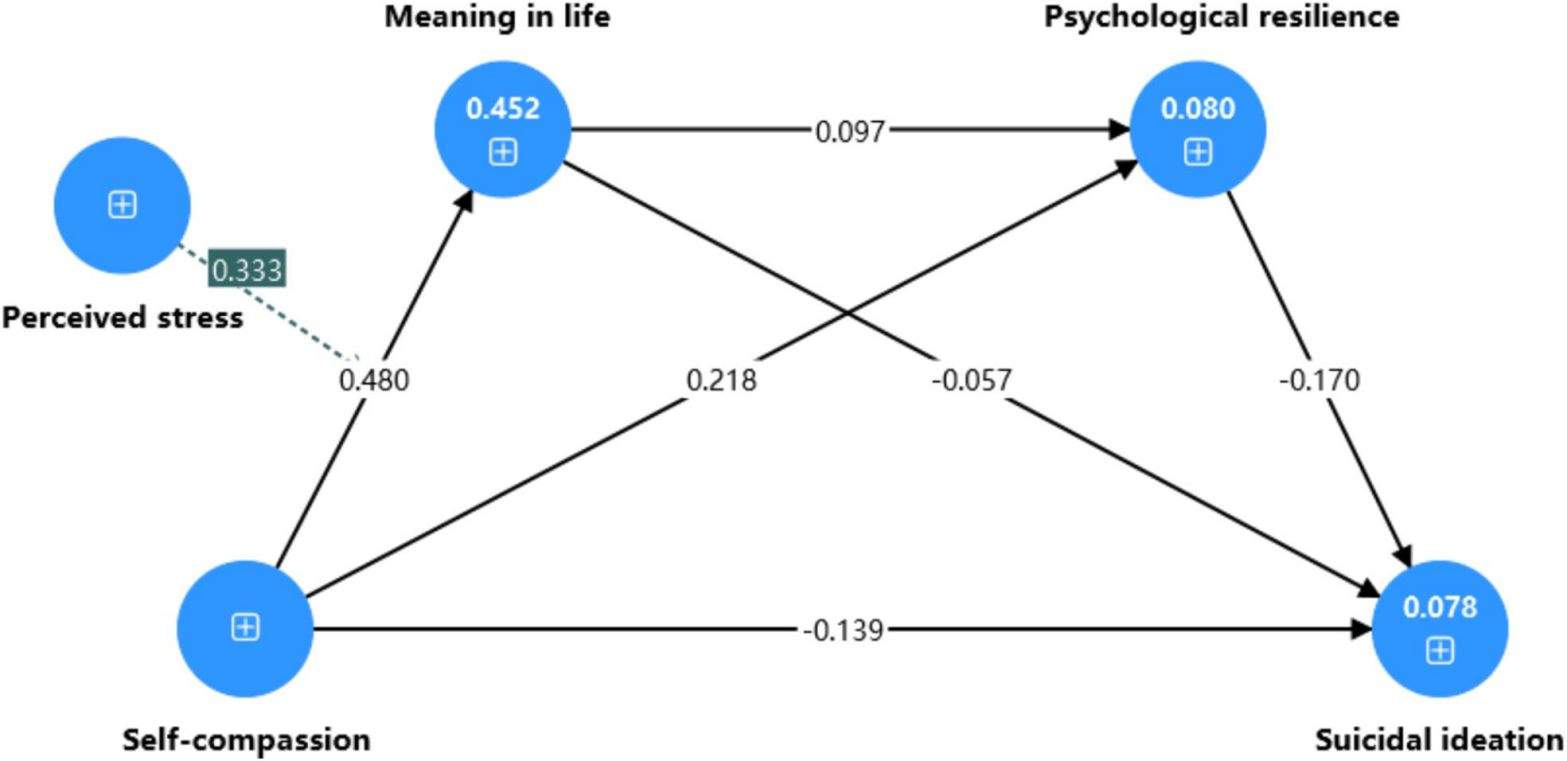
Moderated mediation effect model.

According to the PLS findings, higher self-compassion corresponded to greater MIL and psychological resilience, and lower suicidal ideation. Higher perceived stress aligned with lower MIL. Greater MIL was linked to stronger psychological resilience, and stronger psychological resilience was linked to reduced suicidal ideation. No significant direct link appeared between MIL and suicidal ideation (Figure 2; Table 8).

**Table 8:** Path hypothesis testing.

Hypothesis	Original sample	2.50%	97.50%	T	*p*	Results
SC → SI	−0.139	−0.232	−0.043	2.905	0.004	Supported
SC → MIL	0.480	0.431	0.529	18.938	<0.001	Supported
SC → PR	0.218	0.141	0.300	5.390	<0.001	Supported
MIL → SI	−0.057	−0.141	0.022	1.400	0.162	Unsupported
MIL → PR	0.097	0.017	0.176	2.402	0.016	Supported
PR → SI	−0.170	−0.240	−0.107	5.061	<0.001	Supported

#### 4.5.2 Coefficient of determination

To measure how well the model fits the data and predicts new observations, we calculated the determination coefficient (*R*^2^) and the predictive relevance statistic (*Q*^2^) for the constructs that are affected by other variables in the model, applying guidelines from Hair et al. (2021). For suicidal ideation, *R*^2^ was 0.182, so the predictors accounted for 18.2% of its variance. All *Q*^2^ values were above zero (Table 9), which meets the usual standard for acceptable predictive relevance. The global fit indices also fell within an acceptable range: SRMR was 0.033 (< 0.05), and NFI was 0.897, slightly below the often cited 0.90 cut-off but still consistent with an overall adequate fit.

**Table 9:** Explanatory power and predictive relevance.

Constructs	*R* ^2^	*Q* ^2^	Model fit
SI	0.182	0.046	SRMR: 0.033 NFI: 0.897

“*R*^2^” represents the explanatory power of the model, “*Q*^2^” indicates the predictive relevance of the model.

### 4.6 Mediation analysis

We used a bootstrapping approach (Teo et al., 2015) with 5,000 resamples to examine whether MIL and psychological resilience carry indirect paths to suicidal ideation. Table 10 reports the detailed effects. Self-compassion showed a negative indirect path to suicidal ideation through the serial chain MIL → psychological resilience and another negative indirect path through psychological resilience alone. MIL also showed a negative indirect path to suicidal ideation through psychological resilience, while the direct path from MIL to suicidal ideation was not significant. Overall, these results point to a serial mediation pattern in which MIL and psychological resilience stand between self-compassion and suicidal ideation.

**Table 10:** Mediating analysis.

Relationship	Indirect effect	2.5%	97.5%	T	*p*	Direct effect	T	*p*	Mediation type
SC → MIL → SI	−0.028	−0.006	0.057	1.396	0.163	−0.139	2.905	0.004	NA
SC → PR → SI	−0.037	−0.061	−0.020	3.539	<0.001	−0.139	2.905	0.004	CPM
MIL → PR → SI	−0.017	−0.033	−0.003	2.106	0.035	−0.057	1.400	0.162	FM
SC → MIL → PR → SI	−0.008	−0.016	−0.001	2.063	0.039	−0.139	2.905	0.004	CPM

“FM” indicates full mediation; “CPM” denotes partial mediation; “NA” signifies not applicable.

### 4.7 Moderating analysis

The moderating role of perceived stress was examined in Smart PLS (Becker et al., 2012). The product term of self-compassion and perceived stress showed a positive association with MIL (Table 11), meaning that higher levels of perceived stress intensified the connection of self-compassion with MIL.

**Table 11:** Moderating analysis.

Path	*β*	2.5%	97.5%	T	*p*	Result
PS × SC → MIL	0.333	0.276	0.380	12.720	<0.001	Supported

## 5 Discussion

We examined self-compassion and suicidal ideation in college students and used MIL and psychological resilience as mediators and perceived stress as a moderator in the model. Our results indicate that self-compassion and suicidal ideation show connections both directly and through a serial pathway that passes through MIL and psychological resilience. Perceived stress stands out as a key context for this pattern. All hypotheses matched the empirical results except the one that proposed MIL as a single mediator between self-compassion and suicidal ideation. Later in this paper, we look at these hypotheses and their corresponding results in greater depth.

Data from this study reveal that greater self-compassion corresponds to lower levels of suicidal ideation, which supports *H1*. This pattern matches earlier studies (Umphrey et al., 2021; Zhang et al., 2025), where greater self-compassion scores are consistently linked to diminished suicidal ideation. In our college student sample, those with greater self-compassion also reported fewer thoughts of suicide. One way to understand this pattern is to look at the emotional style that goes together with self-compassion. Strong suicidal ideation often comes with intense self-criticism and self-attacking tendencies (Zhang et al., 2025). Self-compassion invites students to treat themselves with kindness and acceptance (Neff, 2003), to notice their own suffering without harsh judgment, and to turn inner hostility into self-support. The negative correlations observed between self-compassion and suicidal ideation fit well with this more caring attitude toward the self.

*H2* did not gain support. No mediating role was found for MIL alone between self-compassion and suicidal ideation. The data still revealed that students with higher self-compassion typically reported stronger MIL, in agreement with earlier results (He et al., 2025; Wu et al., 2022). The main discrepancy concerned MIL and suicidal ideation. In our data, the path from MIL to suicidal ideation did not reach significance, whereas Diao et al. (2025) found a significant negative link between these two variables. Reported a significant negative association. One possible explanation lies in sample differences. In sequential models, psychological resilience may be a closer and stronger predictor, rendering the distal effect of MIL statistically redundant. Specifically, psychological resilience may play a role at an early stage as a significant buffer early on, protecting against the onset of suicidal ideation. Given its close association with coping strategies and emotion regulation, psychological resilience may have a more significant direct correlation with suicidal ideation, while the role of MIL is more indirect. Therefore, psychological resilience may account for a large percentage of the variance in suicidal ideation, thus overshadowing the role of MIL in this process. In addition, sample differences may still contribute to this variation. Our sample consists of undergraduate students from different disciplines, whose sources of stress and value systems are more diverse. For these students, MIL may not serve as a significant psychological barrier in the same direct way it does for graduate students in high-pressure fields like medicine.

*H3* received support. Psychological resilience mediated the connection from self-compassion to suicidal ideation. This finding fits prior work (Shu et al., 2024; Yang Y. M. et al., 2023) and points to a pattern in which high self-compassion and strong psychological resilience often appear together with lower suicidal ideation. As a core capacity for coping with adversity, psychological resilience may help college students with higher self-compassion translate a kind and accepting attitude toward the self into sustained adaptive capacity when facing setbacks, thereby more effectively buffering suicidal ideation. This finding also helps to explain why the mediating role of MIL alone was not significant. Compared with the relatively abstract construct of MIL, psychological resilience is more closely tied to emotion regulation and stress coping and may therefore play a more direct mediating role in the proximal psychological processes related to suicidal ideation.

Notably, MIL and psychological resilience together formed a serial mediating route from self-compassion to suicidal ideation, so *H4* received support. This result fits earlier work: self-compassion shows positive link to MIL (Chan et al., 2022; He et al., 2025), MIL shows positive associations with psychological resilience (Guo et al., 2025), and higher psychological resilience shows negative associations with suicidal ideation (Chen et al., 2023; Shu et al., 2024). Stated differently, one part of the link between self-compassion and suicidal ideation runs through a sequence in which higher self-compassion goes together with stronger MIL and, in turn, higher psychological resilience.

The findings for *H5* show a clear moderation pattern. Earlier work by Neff and McGehee (2009) supports this pattern, as they reported that self-compassion works most clearly as a shield in high-stress contexts. COR theory (Hobfoll, 1989) points out that long-lasting stress drains students’ inner resources. When students juggle exams, coursework and plans for future jobs, many of them turn to self-compassion as a personal tool for coping, easing harsh inner talk and calming strong emotions. This way of thinking helps them continue to see purpose and MIL during challenging times. In this way, perceived stress did not weaken what self-compassion does; instead, it made the connection linking self-compassion with MIL more apparent.

## 6 Impact

### 6.1 Theoretical impact

From a theoretical angle, our work adds two main points. One point is the chain “MIL → psychological resilience,” which brings together two often studied protective factors and places them inside a single process pattern that connects self-compassion and suicidal ideation. The second point concerns stress: our results show that the links in this chain differ across perceived stress, and this pattern broadens the use of COR theory in studies of stress and meaning building and highlights the context dependent side of psychological protection.

### 6.2 Practical impact

From a practical view, this work points to several steps universities can take when they design suicide prevention for students. One step is to bring self-compassion, MIL, and psychological resilience into campus mental health education, for instance by offering self-compassion workshops, small group counseling, and exercises that train mindful awareness and kinder inner speech. Based on the path “self-compassion → MIL → psychological resilience,” group work with students facing elevated risks can follow a clear order: start with self-compassion, move on to questions of life meaning, and then focus on building resilience skills. Because the connection between self-compassion and MIL appears stronger when perceived stress is high, brief skill trainings such as simple breathing and grounding exercises may be most timely in intense periods like exam weeks or thesis defenses.

At the individual level, college students can cultivate self-compassion and related resources through small, manageable practices. Short daily exercises, such as briefly noting a difficult event, recognizing self-critical thoughts, and consciously reframing them in a more understanding and supportive way, may help foster a habit of self-support in times of distress. Regular involvement in personally meaningful or prosocial activities can also strengthen MIL and, in turn, psychological resilience. Finally, when students face acute stress, they may benefit from a simple self-compassion exercise that can be used in the moment: first deliberately pausing and noticing their emotional state, then taking several slow, deep breaths, and finally saying a brief, supportive phrase to themselves (e.g., “This is hard, but I can take it one step at a time”). This sequence can help reduce emotional overload and foster more adaptive coping.

### 6.3 Limitations and future directions

Our work is only a first step and comes with several limits. The cross-sectional design means that we only see one time point, so we cannot tell which change comes first. Future projects can follow students over several waves and use longitudinal models to map how the main variables move across time. We also relied mostly on self-report. Later studies can mix in ecological momentary assessment, short daily surveys, and simple behavioral tasks, so that day-to-day feelings and actions are covered in a richer way. Another direction is to sharpen the core constructs. Researchers can separate academic stress, family stress, and social stress, look at their different roles in the model, and bring in cultural and family background to build a more ecological picture of students’ lives. The final step is practice. Future researchers can design group programs based on this framework, test them in randomized controlled trials, and see how well such programs work in real campus settings.

## 7 Conclusion

This study focused on college students and brought self-compassion, suicidal ideation, MIL, psychological resilience and perceived stress into one framework. In our model, a sequential pathway through MIL and psychological resilience connected self-compassion to suicidal ideation. Perceived stress also moderated the relationship of self-compassion with MIL. Using self-report questionnaires from 905 students, we applied PLS-SEM to test this model. Higher self-compassion coincided with lower suicidal ideation, and part of this pattern went through MIL and psychological resilience. Perceived stress moderated the link between self-compassion and MIL. Because the data came from one cross-sectional survey in Chinese universities, the conclusions may not carry over to other settings, and the order of change among the variables still needs further work. Subsequent research should aim to validate the model through two key approaches: first, by adopting longitudinal or experimental methodologies; second, by examining its applicability in diverse cultural settings. These findings add to knowledge about how campus protective factors connect with suicidal ideation and offer ideas for future mental health programs.

## Data Availability

The datasets presented in this study can be found in online repositories. The names of the repository/repositories and accession number(s) can be found in the article/supplementary material.

## References

[ref1] Arslan G. Yildirim M. (2021). A longitudinal examination of the association between meaning in life, resilience, and mental well-being in times of coronavirus pandemic. Front. Psychol. 12:645597. doi: 10.3389/fpsyg.2021.645597, 33995201 PMC8113805

[ref2] Beck A. T. Kovacs M. Weissman A. (1979). Assessment of suicidal intention: the scale for suicide ideation. J. Consult. Clin. Psychol. 47, 343–352. doi: 10.1037/0022-006X.47.2.343 469082

[ref3] Becker J.-M. Cheah J.-H. Gholamzade R. Ringle C. M. Sarstedt M. (2022). PLS-SEM’S most wanted guidance. Int. J. Contemp. Hosp. Manag. 35, 321–346. doi: 10.1108/ijchm-04-2022-0474

[ref4] Becker J.-M. Klein K. Wetzels M. (2012). Hierarchical latent variable models in PLS-SEM: guidelines for using reflective-formative type models. Long Range Plan. 45, 359–394. doi: 10.1016/j.lrp.2012.10.001

[ref5] Bryan C. J. Allen M. H. Wastler H. M. Bryan A. O. Baker J. C. May A. M. . (2023). Rapid intensification of suicide risk preceding suicidal behavior among primary care patients. Suicide Life Threat. Behav. 53, 352–361. doi: 10.1111/sltb.12948, 36912126

[ref6] Bryan C. J. Rudd M. D. (2023). Suicidal ideation and suicidal beliefs as prospective indicators of suicidal behavior among primary care patients. Psychiatry Res. Commun. 3:100107. doi: 10.1016/j.psycom.2023.100107

[ref7] Chan K. K. S. Lee J. C. K. Yu E. K. W. Chan A. W. Y. Leung A. N. M. Cheung R. Y. M. . (2022). The impact of compassion from others and self-compassion on psychological distress, flourishing, and meaning in life among university students. Mindfulness 13, 1490–1498. doi: 10.1007/s12671-022-01891-x 35506030 PMC9050348

[ref8] Chen H. M. Liu Y. F. Qi T. A. Qin Z. X. Zheng S. T. Guo S. R. (2025). Exploring the influence of college students' crisis life events on suicidal ideation: a chain multiple mediation model linking social support, anxiety and depression. Curr. Psychol. 44, 15021–15033. doi: 10.1007/s12144-025-08242-8

[ref9] Chen W. Tian X. Zhang G. Liu J. Shouying Z. (2021). Reliability and validity of the perceived stress scale short form (PSS10) for Chinese college students. Psychol. Explor. 41, 343–348.

[ref10] Chen Y. L. Xie R. B. Tan D. Q. Wang X. Y. Fan R. T. Li W. J. . (2023). Bidirectional longitudinal relationships between victimization, resilience and suicidal ideation of adolescents. Child Youth Serv. Rev. 154:107130. doi: 10.1016/j.childyouth.2023.107130

[ref11] Cheng M. M. Lian X. Zhang H. P. (2024). Stress and suicidal ideation among Chinese college students: the role of meaning in life. Death Stud. 48, 1121–1128. doi: 10.1080/07481187.2024.2305336, 38236992

[ref12] Diao R. Yao J. Li J. (2025). The impact of digital stress on suicide ideation among medical postgraduates: the chain mediating role of emotion regulation and meaning in life. BMC Psychol. 13:1246. doi: 10.1186/s40359-025-03534-x, 41219947 PMC12607066

[ref13] DiFonte M. C. Schick M. R. Spillane N. S. (2022). Perceived stress and resilience among college students: the roles of self-compassion and anxiety symptomatology. J. Am. Coll. Heal. 72, 128–134. doi: 10.1080/07448481.2021.2024211, 35114902

[ref14] Dizon J. Mendoza N. B. (2023). Low perceived social rank increases the impact of mental health symptoms on suicidal ideation: evidence among young adults from the Philippines. Arch. Suicide Res. 27, 522–539. doi: 10.1080/13811118.2021.2022050, 34989659

[ref15] Djajadisastra F. W. Ma J. S. Musabiq S. Geshica L. (2025). Relationship between self-compassion, thwarted interpersonal needs, and suicidal thoughts among Indonesian young adults. Mindfulness 16, 1002–1014. doi: 10.1007/s12671-025-02540-9

[ref16] Frankl V. E. (1962). Man’s search for meaning: an introduction to logotherapy. Boston, MA: Beacon Press.

[ref17] Frankl V. E. (2006). Man's search for meaning. Boston, MA: Beacon Press.

[ref18] Gilbert P. (2005). Compassion: Conceptualisations, research and use in psychotherapy. 1st Edn. London, England: Routledge. doi: 10.4324/9780203003459

[ref19] Guo J. Guo B. Ren J. Wu M. Liu J. (2025). A serial mediation model of failure mindset and suicidal ideation among undergraduate students: the impact of meaning in life and spiritual coping. Acta Psychol. 255:104954. doi: 10.1016/j.actpsy.2025.104954 40157025

[ref20] Hair J. F. Hult G. T. M. Ringle C. M. Sarstedt M. (2021). A primer on partial least squares structural equation modeling (PLS-SEM). 3rd Edn. Thousand Oaks, CA: SAGE Publications, Inc.

[ref21] He Q. Chen Y. Cao S. (2025). The effect of psychological richness on the meaning in life of college students: the chain mediating effect of sense of coherence and self-compassion. Front. Psychol. 16:1664328. doi: 10.3389/fpsyg.2025.1664328, 41143022 PMC12546130

[ref22] Heine S. J. Proulx T. Vohs K. D. (2006). The meaning maintenance model: on the coherence of social motivations. Personal. Soc. Psychol. Rev. 10, 88–110. doi: 10.1207/s15327957pspr1002_1 16768649

[ref23] Hirsch J. K. Hall B. B. Wise H. A. Brooks B. D. Chang E. C. Sirois F. M. (2021). Negative life events and suicide risk in college students: conditional indirect effects of hopelessness and self-compassion. J. Am. Coll. Heal. 69, 546–553. doi: 10.1080/07448481.2019.1692023, 31765290

[ref24] Hobfoll S. E. (1989). Conservation of resources: a new attempt at conceptualizing stress. Am. Psychol. 44, 513–524. doi: 10.1037/0003-066X.44.3.513 2648906

[ref25] Hu L. T. Bentler P. M. (1999). Cutoff criteria for fit indexes in covariance structure analysis: conventional criteria versus new alternatives. Struct. Equation Modell. Multidiscip. J. 6, 1–55. doi: 10.1080/10705519909540118

[ref26] Hussain Z. Hill R. M. (2023). The association between coping behaviors and the interpersonal theory of suicide in college students. J. Behav. Cognit. Ther. 33, 118–126. doi: 10.1016/j.jbct.2023.05.005

[ref27] Jiang M. Ding W. Feng J. Wang X. Xie R. (2024). Developmental relations between depression and suicidal ideation among adolescents: a latent change score modeling study. Pers. Individ. Differ. 230:112772. doi: 10.1016/j.paid.2024.112772

[ref28] Kato T. (2025). Effects of self-compassion on depressive symptoms and suicide risk in college students: a longitudinal study. J. Affect. Disord. 390:119840. doi: 10.1016/j.jad.2025.119840, 40633780

[ref29] Keulen J. Deković M. Vervoort L. Bodden D. (2025). Exploring transdiagnostic factors of internalizing and externalizing symptoms in transitional-age youth: using a variable-centered and person-centered approach. Curr. Psychol. 44, 16866–16882. doi: 10.1007/s12144-025-08328-3, 41199989 PMC12586208

[ref30] Kim B. J. Kihl T. (2021). Suicidal ideation associated with depression and social support: a survey-based analysis of older adults in South Korea. BMC Psychiatry 21:409. doi: 10.1186/s12888-021-03423-8, 34407801 PMC8375215

[ref31] Kirshenbaum J. S. Pagliaccio D. Bitran A. Xu E. Auerbach R. P. (2024). Why do adolescents attempt suicide? Insights from leading ideation-to-action suicide theories: a systematic review. Transl. Psychiatry 14:266. doi: 10.1038/s41398-024-02914-y, 38937430 PMC11211511

[ref32] Klonsky E. D. May A. M. (2015). The three-step theory (3ST): a new theory of suicide rooted in the "ideation-to-action" framework. Int. J. Cogn. Ther. 8, 114–129. doi: 10.1521/ijct.2015.8.2.114

[ref33] Kumar S. A. Jaffe A. E. Brock R. L. DiLillo D. (2022). Resilience to suicidal ideation among college sexual assault survivors: the protective role of optimism and gratitude in the context of posttraumatic stress. Psychol. Trauma Theory Res. Pract. Policy 14, S91–S100. doi: 10.1037/tra0001141, 34591537 PMC8930426

[ref34] Lasota A. Mróz J. (2021). Positive psychology in times of pandemic-time perspective as a moderator of the relationship between resilience and meaning in life. Int. J. Environ. Res. Public Health 18:Article 13340. doi: 10.3390/ijerph182413340, 34948949 PMC8707410

[ref35] Leguina A. (2015). A primer on partial least squares structural equation modeling (PLS-SEM). International Journal of Research & Method in Education, 38, 220–221. Oxford, England: Taylor & Francis. doi: 10.1080/1743727X.2015.1005806

[ref36] Liang M. Xu H. J. Jiang Q. Liu T. S. (2025). Psychological pain tolerance mediates the association between physical pain sensitivity and suicidal ideation: a cross-sectional study. BMC Psychiatry 25:692. doi: 10.1186/s12888-025-07130-6, 40640759 PMC12243396

[ref37] Liu J. Dang J. H. Zou H. Y. (2025). Exploring the longitudinal dynamics of self-esteem, body image, and psychological resilience in college students. J. Pac. Rim Psychol. 19:18344909251360073. doi: 10.1177/18344909251360073

[ref38] Liu M. Yu X. Liu A. Wu X. Lin W. (2025). Childhood maltreatment and suicide ideation in Chinese college students: the chain mediating role of self-compassion and depressive symptoms. Child Abuse Negl. 165:107475. doi: 10.1016/j.chiabu.2025.107475, 40311487

[ref39] Low Y. S. Bhar S. S. Chen W. S. (2023). Moderators of suicide ideation in Asian international students studying in Australia. Aust. Psychol. 58, 169–178. doi: 10.1080/00050067.2022.2148514

[ref40] Luceño-Moreno L. Vázquez-Estévez D. Martín-García J. Talavera-Velasco B. Bosurgi R. (2025). Factors associated with suicidal ideation in college students of health sciences. Depress. Anxiety 2025:4397417. doi: 10.1155/da/4397417 PMC12321416 40761832

[ref41] Ma Z. J. Xu T. T. Ma C. H. Li D. W. Zheng D. W. Sun X. Y. (2024). Predictive effect of social support on posttraumatic growth of medical students in China. Soc. Behav. Pers. 52:e13560. doi: 10.2224/sbp.13560

[ref42] Mao Y. X. Liu L. M. Lu Z. Wang W. C. (2022). Relationships between perceived discrimination and suicidal ideation among impoverished Chinese college students: the mediating roles of social support and loneliness. Int. J. Environ. Res. Public Health 19:7290. doi: 10.3390/ijerph19127290, 35742540 PMC9223897

[ref43] Marrero R. J. Bello E. M. Morales-Marrero D. Fumero A. (2024). Distinguishing the role of adverse life events in suicidality and non-suicidal self-injury in Spanish adolescents and young adults. Curr. Psychol. 43, 22321–22332. doi: 10.1007/s12144-024-05883-z

[ref44] Miao M. Wen J. Cao R. Jin S. Gan Y. (2025). Self-compassion among Chinese adolescents: internal structure, latent profiles, and its longitudinal association with meaning in life. Mindfulness 16, 2586–2601. doi: 10.1007/s12671-025-02649-x

[ref45] Neff K. (2003). Self-compassion: an alternative conceptualization of a healthy attitude toward oneself. Self Identity 2, 85–101. doi: 10.1080/15298860309032

[ref46] Neff K. D. McGehee P. (2009). Self-compassion and psychological resilience among adolescents and young adults. Self Identity 9, 225–240. doi: 10.1080/15298860902979307

[ref47] Osman A. Barrios F. X. Gutierrez P. M. Wrangham J. J. Kopper B. A. Truelove R. S. . (2002). The positive and negative suicide ideation (PANSI) inventory: psychometric evaluation with adolescent psychiatric inpatient samples. J. Pers. Assess. 79, 512–530. doi: 10.1207/S15327752JPA7903_07, 12511018

[ref48] Peng X. F. Tang T. G. Wu M. Tan L. Pan Y. G. (2024). Network analysis of risk and protective factors for suicidal ideation in adolescents. Child Youth Serv. Rev. 158:107458. doi: 10.1016/j.childyouth.2024.107458

[ref49] Podsakoff P. M. MacKenzie S. B. Lee J. Y. Podsakoff N. P. (2003). Common method biases in behavioral research: a critical review of the literature and recommended remedies. J. Appl. Psychol. 88, 879–903. doi: 10.1037/0021-9010.88.5.879, 14516251

[ref50] Premkumar G. Bhattacherjee A. (2008). Explaining information technology usage: a test of competing models. Omega 36, 64–75. doi: 10.1016/j.omega.2005.12.002

[ref51] Ramos-Martín J. Pérez-Berlanga J. M. Oliver J. Moreno-Küstner B. (2023). Non-lethal suicidal behavior in university students of Spain during COVID-19. Frontiers. Psychiatry 14:1155171. doi: 10.3389/fpsyt.2023.1155171, 37533884 PMC10390698

[ref52] Reker G. T. Wong P. P. T. (1988). “Aging as an individual process: toward a theory of personal meaning” in Emergent theories of aging. eds. Birren J. E. Bengtson L. (New York, NY: Springer Publishing Co.).

[ref53] Riera-Serra P. Navarra-Ventura G. Castro A. Gili M. Salazar-Cedillo A. Ricci-Cabello I. . (2024). Clinical predictors of suicidal ideation, suicide attempts and suicide death in depressive disorder: a systematic review and meta-analysis. Eur. Arch. Psychiatry Clin. Neurosci. 274, 1543–1563. doi: 10.1007/s00406-023-01716-5 38015265 PMC11422269

[ref54] Shu Z. Chen S. R. Chen H. Chen X. L. Tang H. J. Zhou J. W. . (2024). The effects of subjective family status and subjective school status on depression and suicidal ideation among adolescents: the role of anxiety and psychological resilience. PeerJ 12:e18225. doi: 10.7717/peerj.18225, 39430566 PMC11488497

[ref55] Steger M. F. Frazier P. Oishi S. Kaler M. (2006). The meaning in life questionnaire: assessing the presence of and search for meaning in life. J. Couns. Psychol. 53, 80–93. doi: 10.1037/0022-0167.53.1.80

[ref56] Suh H. Jeong J. (2021). Association of self-compassion with suicidal thoughts and behaviors and non-suicidal self injury: a meta-analysis. Front. Psychol. 12:633482. doi: 10.3389/fpsyg.2021.633482, 34122224 PMC8192964

[ref57] Tekinarslan F. G. Sevi Tok E. S. (2023). Engelli Çocuğu Olan Annelerde Öz-Şefkat, Sosyal Destek ve Psikolojik Dayanıklılığın Öznel İyi Oluşla İlişkisi. Ayna Klinik Psikoloji Dergisi-Ayna Clinical Psychology Journal 10, 145–164. doi: 10.31682/ayna.1145256

[ref58] Teo A.-C. Tan G. W.-H. Ooi K.-B. Hew T.-S. Yew K.-T. (2015). The effects of convenience and speed in m-payment. Ind. Manag. Data Syst. 115, 311–331. doi: 10.1108/imds-08-2014-0231

[ref59] Umphrey L. R. Sherblom J. C. Swiatkowski P. (2021). Relationship of self-compassion, hope, and emotional control to perceived burdensomeness thwarted belongingness, and suicidal ideation. Crisis 42, 121–127. doi: 10.1027/0227-5910/a000697 32672522

[ref60] Wang X. Li H. (2025). Psychological distress and suicidal ideation in patients with depressive disorders: the chain mediation of psychological resilience and neuroticism. Int. J. Nurs. Stud. Adv. 8:100325. doi: 10.1016/j.ijnsa.2025.100325 PMC12018043 40276211

[ref61] Wang Z. Y. Li C. K. Xie Z. Hong O. L. (2024). Grit difference in the association between academic stress and adolescents' meaning in life: the roles of school burnout and self-compassion. Child Care Health Dev. 50:e70005. doi: 10.1111/cch.70005, 39540694

[ref62] Wang L. Shi Z. Zhang Y. Zhang Z. (2010). Psychometric properties of the 10-item Connor–Davidson resilience scale in Chinese earthquake victims. Psychiatry Clin. Neurosci. 64, 499–504. doi: 10.1111/j.1440-1819.2010.02130.x 20923429

[ref63] Wang Z. Zhu Y. Zhan X. Wang T. Tang X. Li L. . (2024). Problem-solving ability and future time perspective among the Chinese nursing interns: the mediating role of future work self. PLoS One 19:e0308669. doi: 10.1371/journal.pone.0308669, 39116140 PMC11309400

[ref64] World Health Organization (2021). Suicide worldwide in 2019: global health estimates. World Health Organization. Available online at: https://www.who.int/publications/i/item/9789240026643

[ref65] World Health Organization. (2025). Suicide. Geneva, Switzerland: World Health Organization. Available online at: https://www.who.int/news-room/fact-sheets/detail/suicide (Accessed November 17, 2025)

[ref66] Wu S. F. Adamsk K. (2021). Intervention effect of cognitive behaviour therapy under suicidology on psychological stress and emotional depression of college students. Work 69, 697–709. doi: 10.3233/WOR-213510, 34120946

[ref67] Wu N. N. Hou Y. Jiang Y. Q. Zeng Q. You J. N. (2024). Longitudinal relations between social relationships and adolescent life satisfaction: the mediating roles of self-compassion and psychological resilience. J. Child Fam. Stud. 33, 2195–2208. doi: 10.1007/s10826-024-02842-x

[ref68] Wu D. H. Ye B. J. Tang C. Y. Xue J. J. Yang Q. Xia F. (2022). Self-compassion and authentic-durable happiness during COVID-19 pandemic: the mediating role of meaning of life and the moderating role of COVID-19 burnout. Psychol. Res. Behav. Manag. 15, 3243–3255. doi: 10.2147/PRBM.S380874, 36387037 PMC9642803

[ref69] Yang T. Q. He Y. Wu L. Ren L. Lin J. Y. Wang C. X. . (2023). The relationships between anxiety and suicidal ideation and between depression and suicidal ideation among Chinese college students: a network analysis. Heliyon 9:e20938. doi: 10.1016/j.heliyon.2023.e20938, 37876446 PMC10590950

[ref70] Yang Y. M. Liu M. X. Cao N. Li X. Y. Chen Z. S. Kelifa M. O. (2023). School connectedness and psychological resilience as mediators in the relationship between childhood abuse and suicidal ideation among Chinese adolescents. Eur. J. Psychotraumatol. 14:2172650. doi: 10.1080/20008066.2023.2172650, 37052111 PMC9930799

[ref71] Yao Z. Y. Xu X. M. Kou C. G. Wang X. T. Liu B. P. Cheng S. L. . (2025). Comparison of suicidal behavior among Chinese university students before and during the COVID-19 pandemic: findings from a two-wave cross-sectional study. Death Stud. 1-16, 1–16. doi: 10.1080/07481187.2025.2476981, 40068991

[ref72] Yildirim M. Aksoy S. Öztekin G. G. Alkhulayfi A. M. A. Aziz I. A. Gómez-Salgado J. (2025). Resilience, meaning in life, and perceived social support mediate the relationship between fear of happiness and psychological distress. Sci. Rep. 15:34270. doi: 10.1038/s41598-025-16486-4, 41034294 PMC12489059

[ref73] You S. Kwon M. Kim E. K. (2022). Perfectionism, life stress, and suicidal ideation among college students: a protective role of self-compassion. J. Exp. Psychopathol. 13:20438087221103350. doi: 10.1177/20438087221103350

[ref74] Zhang S. N. Ren Y. Z. Zeng H. P. Rong D. H. Ren H. L. Jiang T. Y. . (2025). Mindfulness, perceived social support, and suicidal ideation among Chinese adolescents: the mediating role of self-compassion. Front. Psych. 16:1613442. doi: 10.3389/fpsyt.2025.1613442, 41019594 PMC12461862

[ref75] Zhou X. L. Chen D. R. Wu H. J. Ying J. F. Shen Y. H. Zhu Q. X. . (2023). The protective effect of trait mindfulness on the associations between negative perfectionism and suicidal ideation among Chinese adolescents: a longitudinal moderated mediation model. Mindfulness 14, 395–405. doi: 10.1007/s12671-023-02069-9

[ref76] Zhou J. R. Sun F. (2025). The relationship of physical exercise and suicidal ideation among college students: a moderated chain mediation model. Front. Psychol. 16:1624998. doi: 10.3389/fpsyg.2025.1624998, 40678437 PMC12268211

